# Multiplex Real-Time Polymerase Chain Reaction and Recombinase Polymerase Amplification: Methods for Quick and Cost-Effective Detection of Vancomycin-Resistant Enterococci (VRE)

**DOI:** 10.3390/antibiotics14030295

**Published:** 2025-03-12

**Authors:** Ibukun Elizabeth Osadare, Abdinasir Abdilahi, Martin Reinicke, Celia Diezel, Maximilian Collatz, Annett Reissig, Stefan Monecke, Ralf Ehricht

**Affiliations:** 1 Leibniz Institute of Photonic Technology (IPHT), Member Research Alliance Leibniz Centre for Photonics in Infection Research (LPI), 07745 Jena, Germany; ibukun-elizabeth.osadare@leibniz-ipht.de (I.E.O.); abdinasir.abdilahi@leibniz-ipht.de (A.A.); martin.reinicke@leibniz-ipht.de (M.R.); celia.deizel@leibniz-ipht.de (C.D.); maximilian.collatz@leibniz-ipht.de (M.C.); annett.reissig@leibniz-ipht.de (A.R.); stefan.monecke@leibniz-ipht.de (S.M.); 2 InfectoGnostics Research Campus, 07743 Jena, Germany; 3 Institute of Physical Chemistry, Friedrich-Schiller University, 07743 Jena, Germany

**Keywords:** *Enterococcus* spp., VRE, resistance genes, DNA, PCR, RPA

## Abstract

**Background**/**Objectives**: Vancomycin-resistant enterococci (VRE) are one of the leading causes of antibiotic-resistant infections in the hospital setting worldwide, and this has become a major issue, because most patients infected with this strain are difficult to treat. Multiplex real-time polymerase chain reaction (RT PCR) is an advantageous technique that can amplify multiple targets in a single reaction, and can be used to quickly detect specific targets in VRE within two hours, starting from suspected colonies of bacterial cultures, without sample preparation. **Methods**: In this study, we selected the glycopeptide/vancomycin resistance genes that are most common in clinical settings, *vanA* and *vanB*, in combination with the species markers *ddl_faecium* and *ddl_faecalis* for the most common VRE species—*Enterococcus faecium* and *Enterococcus faecalis*. **Results**: DNA from forty clinical VRE strains was prepared using a fast and economic heat lysis method, and a multiplex real-time PCR assay was optimized and carried out subsequently. The results were in concordance with the results from recombinase polymerase amplification (RPA) of the same VRE samples. **Conclusions**: Multiplex RT PCR and RPA for VRE detection proffers a second method for the confirmation of vancomycin resistance, and it can be developed as a fast screening assay for patients before admission into high-risk settings.

## 1. Introduction

Antimicrobial resistance is a known and growing threat worldwide [1], and the struggle to overcome this dilemma has provoked the implementation of preventive measures, an increase in related research for rapid diagnostics, and the introduction of new/modified effective treatment methods. Fast determination of microbial susceptibility profiles can be paramount in making the right choice for how to treat antimicrobial resistance. Vancomycin-resistant enterococci (VRE), similarly to other antimicrobial-resistant (AMR) groups [2], can be difficult to treat [3]. Enterococci are Gram-positive bacteria that normally live as commensals in the gastrointestinal tract of humans and some animals. Outside the gut, they can cause a wide range of infections, like intra-abdominal, pelvic, and wound infections (especially after an operation). They can also cause bacteremia and endocarditis, mostly in immunocompromised patients [4]. Septicemia caused by VRE is particularly dangerous, with mortality rates reaching up to 58% [5]. The presence of (multi-)resistant microbes like VRE in clinical high-risk settings, like intensive care units (ICUs), oncology wards, and transplantation units, can lead to further morbidity, and even mortality. One study showed that about ten percent of patients admitted to an ICU were colonized with VRE on admission, although an association between colonization and infection was not established [6]. A hospital in Japan also determined the cumulative incidence of VRE infection within the hospital following an outbreak in 2022. It was identified that patients that had previously been hospitalized in areas with reported VRE outbreaks had the highest cumulative incidence among patients who tested positive on admission, followed by patients requiring toilet assistance [7]. A possible persistent mode of transmission is through the hands of health care workers [8]. There is also a possibility of direct VRE transmission from one patient to another [9]. Likewise, VRE in food products derived from animals also poses a potential public health risk if unchecked [10]. For example, the rise in the number of recorded cases of VRE infection in Europe in the 1990s showed how food products can affect public health [11]. VRE occurrence among livestock and humans was linked with the indiscriminate use of avoparcin (a glycopeptide closely related to vancomycin) by farmers as a growth promoter [11,12]. After avoparcin was banned in Europe, there was a notable decrease in the incidence of VRE infections [13].

Enterococci possess intrinsic resistance to several antibiotics, including cephalosporins, monobactams, meropenem, and clindamycin [4,14]. *Enterococcus faecalis* and *Enterococcus faecium* are the most common enterococcal species in humans. The genes *vanA* and *vanB* are the most common glycopeptide resistance markers in clinical isolates of VRE globally [15,16,17,18,19]. Enterococcal strains that have the *vanA* gene are highly resistant to vancomycin and teicoplanin, while enterococcal strains that are positive for the *vanB* gene are resistant to vancomycin only, but are susceptible to teicoplanin [20]. The mechanisms of action of *vanA* and *vanB* are similar, as they promote the synthesis of a peptidoglycan precursor ending in peptidyl-d-alanyl–d-lactate (D-Ala-D-Lac). D-Ala-D-Lac then binds to glycopeptides and lowers the antibiotics’ affinity for peptidyl-d-alanyl-d-alanine (D-Ala-D-Ala) [21]. D-Ala-D-Ala is the membrane-bound lipid precursor of peptidoglycan. Glycopeptide antibiotics work by binding to D-Ala-D-Ala, preventing its incorporation into the vital structural cell wall component [22].

Following culturing of patient samples for 24 to 72 h, when growth of bacterial colonies is observed, they can be identified using biochemical tests or methods like matrix-assisted laser desorption ionization (MALDI) [23], and susceptibility testing can be performed. Molecular diagnostic methods can be employed afterwards to quickly and accurately detect species-specific and resistance genes in enterococcal strains; the detection of these genes is a prerequisite for effective infection control [24,25,26]. Molecular techniques are beneficial for the rapid screening of patients to be admitted into intensive care units (ICUs), cancer wards, transplant units, and other high-risk settings [27]. They can also be used as a tool for the confirmation of glycopeptide/vancomycin resistance in routine laboratory diagnostics. The development and application of molecular diagnostic techniques has been revolutionary in the diagnosis and monitoring of infectious diseases, because it allows for the identification and better characterization of pathogens [28]. Molecular tests can also be used to directly detect genes or gene mutations that are responsible for drug resistance [29].

Polymerase chain reaction (PCR) is a molecular testing technique that was invented in the 1980s [30]. It has been widely used, especially in the field of infectious diseases. It is an enzymatic assay that allows for the thermally synchronized amplification of a specific DNA fragment within a sample DNA template. Every PCR assay consists of primers, nucleotides, DNA polymerase, and the sample DNA. The primers are short DNA molecules with sequences that are complementary to the target DNA, so they indicate the specific DNA product that should be amplified within the sample DNA. The nucleotides are the building blocks, and the major enzyme that links individual nucleotides together to obtain the PCR product is the thermostable DNA polymerase. The PCR products can be visualized either by staining the amplified product with an intercalating dye, or by using enzymatically introduced fluorophores in oligonucleotides that are incorporated using PCR amplification [31]. Only specific amplicons are detected when fluorophores are linked to oligonucleotides, i.e., to primers or to a TaqMan probe. This is a hydrolysis probe designed to bind to a specific sequence of the target DNA, allowing for precise and sensitive detection of the desired nucleic acid. TaqMan probes have a fluorophore at the 5′ end and a quencher at the 3′ end. The probe is cleaved by DNA polymerase during amplification, resulting in the separation of the fluorophore and quencher; this leads to a fluorescence signal. The fluorescence signal in each amplification cycle is proportional to the quantity of target nucleic acid present in the sample [32]. PCR is fast, has high sensitivity and specificity, and is readily available; consequently, it is widely used in clinical diagnostics [33]. Multiplex real-time PCR uses more than one primer pair in amplifying multiple targets at the same time. This is possible because the target probes are labeled with spectrally distinct fluorophores. Therefore, all targets are distinguishable in a combined reaction, based on their different fluorescence emissions. Multiplex PCR assays have been developed and optimized for various pathogens to improve diagnostic capabilities, as well as to reduce time and cost [2]. One of the focal points of this study is the development of a quick diagnostic multiplex PCR assay for VRE.

In 2006, an isothermal nucleic acid amplification technology was developed, called recombinase polymerase amplification (RPA) [34]. This technology basically involves combining the recombinant enzyme UvsX and RPA primers in the presence of adenine triphosphate (ATP) and polyethylene glycol, to form a recombinase–primer complex. This complex then locates (if present) a complementary sequence in the sample deoxyribonucleic acid (DNA) template, and inserts itself into the template chain to form a D-ring compound that begins a chain replacement reaction. The replaced template is bound to a single-stranded binding protein to prevent expulsion of the inserted primer by chain migration. The recombinase is then isolated from the complex, and DNA polymerase binds to the 3′-OH end of the primer, in the presence of deoxynucleotide triphosphates (dNTPs), for the formation of a new complementary chain through chain elongation. It should be noted that DNA polymerase must have a strand displacement activity for amplification to occur. These steps continue to repeat until a measurable amplification of the target region is achieved. Agarose gel electrophoresis can be used to visualize the RPA amplification product [33,34]. The RPA reaction has a short amplification time, and it occurs at constant incubation temperatures (no thermal synchronization), within a range of 37–42 °C. This means that no expensive cycling device is required, no pre-heating step is required, and the amplification process tolerates more mismatches in primer binding regions. However, there are also disadvantages, like the high cost of reagents and the current availability of only a few commercial kits. This renders the technology unfeasible for diagnostics on a large scale, and thus, it is only used for research purposes [33].

In this study, a multiplex PCR assay was developed and optimized for the detection and/or identification of VRE from suspected VRE colonies without complex kits and sample preparation. An RPA assay for VRE was re-established from pre-existing studies as a control, and the results of both reactions were compared. The multiplex PCR-VRE assay and the RPA reaction assay can be used directly as a screening method for VRE colonization in patients, or implemented into molecular point-of-care assays. They can also be used as an independent method for confirming a culture-based susceptibility test result.

## 2. Results

### 2.1. Multiplex PCR Primer Design

The primers and probes used in this study for the multiplex PCR assay were designed using the ConsensusPrime pipeline, consistently with an approach employed in a prior study [35,36]. High-quality annotated sequence data for enterococcal strains were sourced from the Pathosystems Resource Integration Center (PATRIC) database (www.patricbrc.org, accessed on 21 September 2021). The design was based on multiple sequence alignments of homologue sequences, to guarantee ideal bindings for a great variety of strains. Biophysical parameters like sequence length, melting temperatures, and GC content were considered in the ConsensusPrime pipeline, to amplify all targets under the same experimental conditions. The designed primers and probes (see Table 1) were then synthesized by Metabion International AG (Planegg, Germany), incorporating specific dyes for each probe: *ddl_faecalis* with FAM, *ddl_faecium* with ROX, *vanA* with ATTO 647N (Cy5), and *vanB* with ATTO 550. Primer sequences for the RPA reactions were taken from a published study (see below, Section 4.1), and also synthesized by Metabion International AG.

### 2.2. Optimization of Multiplex PCR for VRE

**Figure 1:**
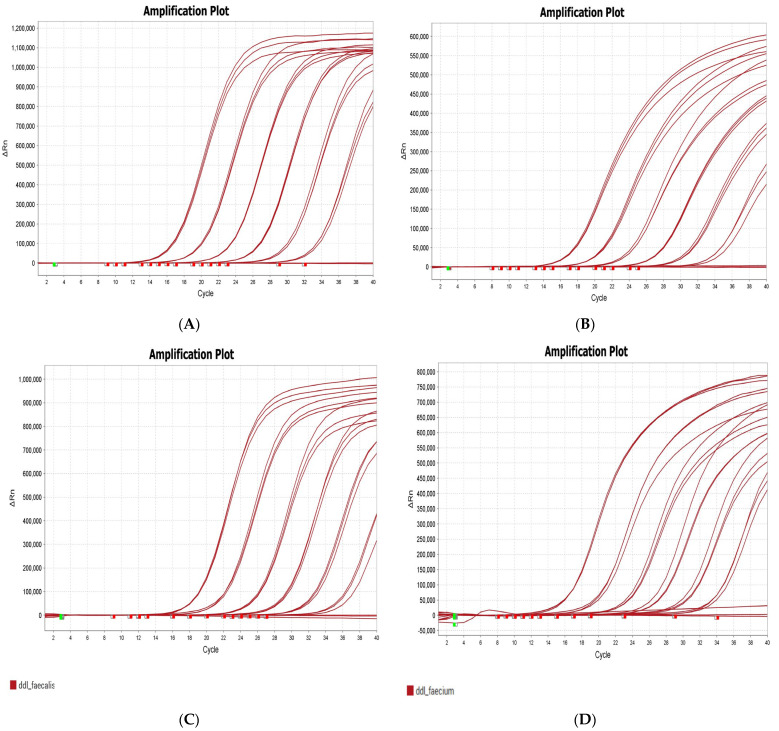
Amplification plots for *vanA* shown in (**A**), *vanB* in (**B**), *ddl_faecalis* in (**C**), and *ddl_faecium* in (**D**). The green squares are the baseline start wells while the red squares are the baseline end wells. Data associated with these amplification plots can be seen in Table 2 and Supplemental File S1.

Single-plex PCR (i.e., with primers for one target in a run) was initially carried out using the designed primers and probes for each of the four targets, to calibrate the PCR curves and to determine the efficiency of the curves (see Table 2, Figure 1, and Supplemental File S1). The PCR runs were optimized from a “single-plex” to a multiplex PCR (with primers for all four targets combined in one run). The total volume of the master mix remained the same, with an equivalent reduction in the volume of nuclease-free water for each additional primer and DNA volume. The QuantStudio 5 (Applied Biosystems by Thermo Fisher Scientific, Bremen, Germany) features four channels with different fluorescence excitation and emission wavelengths, allowing for the detection of the unique dyes attached to each different target probe.

### 2.3. Multiplex PCR Results

The results include color-coded amplification plots representing each target. Targets that were absent were not amplified, and have ∆Rn = 0 in the amplification plot. One experiment was carried out with a mix of DNA isolated from both species, *Enterococcus faecalis* and *Enterococcus faecium*, that had the resistance markers *vanA* and *vanB*.

**Figure 2:**
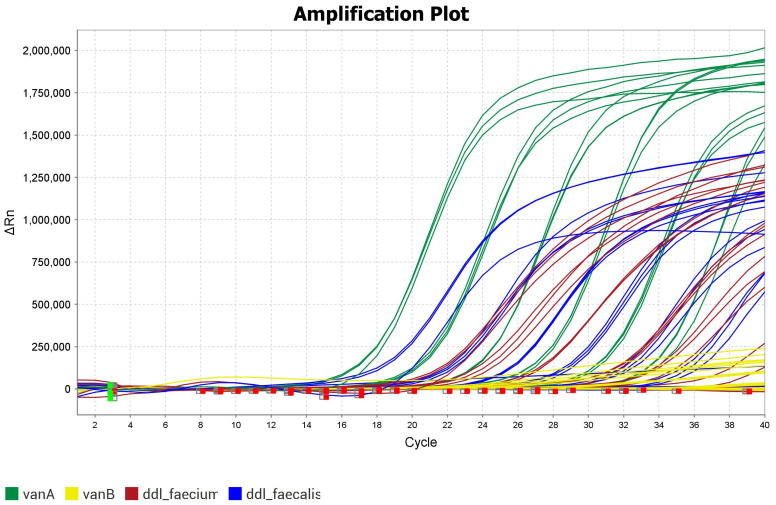
Example of amplification plot for multiplex PCR experiment with all four markers present. Linear plot representing *vanA* is green, that for *vanB* is yellow, and those for *ddl_faecium* and *ddl_faecalis* are red and blue, respectively. The green squares are the baseline start wells and the red squares are the baseline end wells.

This was performed in order to ascertain whether all targets could indeed be amplified at the same time (see Figure 2). The lift for each target, when combined, showed some changes compared to those in their respective calibration curves (Figure 1). However, the targets could be clearly distinguished.

**Figure 3:**
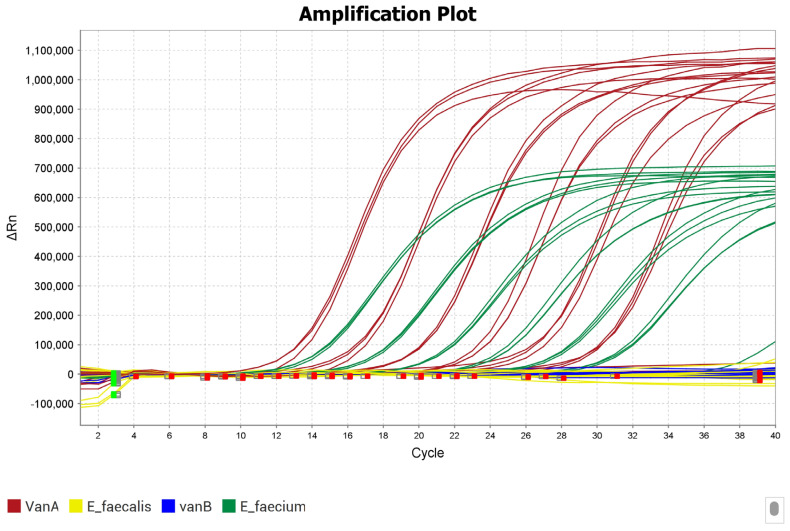
Amplification plot for multiplex PCR results, showing red color-coded *vanA* and green color-coded *ddl_faecium* linear plots for all dilutions. *ddl_faecalis* and *vanB* genes are color-coded yellow and blue in straight lines on zero for all dilutions, including all negative controls. The green squares are the baseline start wells and the red squares are the baseline end wells.

A total of 40 enterococcal strains were analyzed using the multiplex PCR assay. Targets that were present have amplification curves with lift between 500,000 and 1,200,000 for all dilutions. Absent targets have no lift, and appear as straight lines on 0 (see Figure 3). A summary of all the results is provided in Supplemental File S2.

### 2.4. Comparison Between Multiplex PCR and RPA

**Figure 4:**
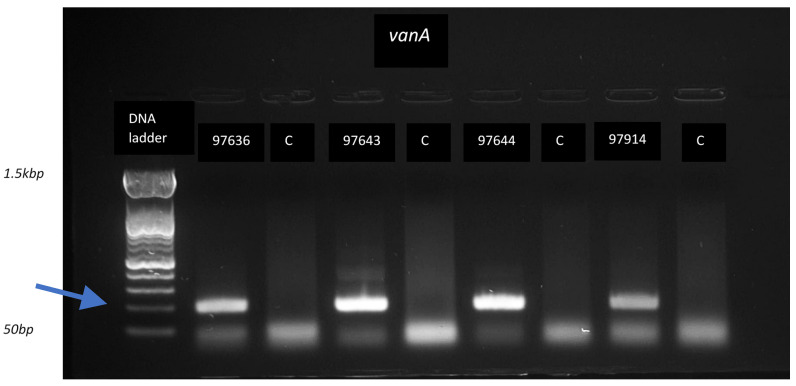
Gel electrophoresis image of RPA product for three different *vanA*-positive *Enterococcus faecalis* experiments (97636, 97643, 97644), one *vanA*-positive *Enterococcus faecium* experiment (97914), and the control experiments (C). *vanA* was amplified in all four strains, as shown by presence of amplification product corresponding to about two hundred base pairs (see arrow).

The amplification products post-RPA were detected using gel electrophoresis (see Figure 4); these results are provided in Supplemental File S3. Two targets were amplified in 20 enterococcal strains using RPA, thus confirming the presence of the targets amplified with multiplex PCR in the same enterococcal strains.

Six of the VRE strains yielded PCR signals for *ddl_faecalis* (Table 3), while the remaining fourteen were positive for *ddl_faecium*. Fourteen of the strains were positive for *vanA*, while six were positive for *vanB*. All *Enterococcus faecalis* strains were positive for the *vanA* resistance gene. The RPA products post-gel electrophoresis showed that six of the VRE strains were positive for *rpoA_faecalis*, thus confirming that the strains were indeed *Enterococcus faecalis.* Fourteen of the VRE strains were positive for *ddl_faecalis*, six were positive for *vanB*, and the remaining fourteen were positive for *vanA*, just as was seen with the results from multiplex PCR. The multiplex RT PCR results were also in concordance with results from previous characterization of the same *Enterococcus faecium* strains by whole genome sequencing (WGS) and DNA microarray for VRE [3], and *Enterococcus faecalis* strains by DNA microarray.

Additionally, the diagnostic sensitivity and specificity for each target were calculated for the 40 enterococcal strains. The diagnostic sensitivity and specificity for *ddl_faecium*, *ddl_faecalis*, and *vanA* were both 100%, while the sensitivity for *vanB* was 96%, and the specificity was 100%. An overview of these results can be seen in Supplemental File S2.

The differences between multiplex RT PCR assay, RPA assay and characterization methods like whole genome sequencing and DNA microarray are highlighted in Table 4.

### 2.5. Heat Lysis of VRE Isolates After Cell Culture

Enterococcal strains were cultured on blood agar plates and used directly for multiplex RT-PCR, following heat lysis (see Section 4.3) of the cells. This minimal sample preparation saved time, and an experiment post-cell culture could be completed within two hours. It also saved costs, as additional materials for DNA purification were not required.

## 3. Discussion

To this day, antimicrobial resistance persists worldwide, and there is ongoing research geared towards finding faster and more accurate diagnostic techniques. Molecular diagnostic methods are specific and accurate, and great strides are being made with this technology, especially in the field of microbiology. Hence, multiplex PCR was the focus in this study, which aimed to finetune an assay that works for detecting the most common target genes in VRE strains.

The aim of this study, which was to optimize a multiplex RT PCR assay for the resistance genes *vanA* and *vanB* and the species markers *ddl_faecium* and *ddl_faecalis* (*Enterococcus faecium* and *Enterococcus faecalis*), which are the most prevalent VRE strains in clinical settings worldwide, from cultured colonies, was achieved. The diagnostic sensitivity and specificity for *ddl_faecalis*, *ddl_faecium*, and *vanA* were 100%, while *vanB* had a specificity of 100% and a sensitivity of 96% for all 40 strains (see Supplemental File S2). This means that the results obtained from the multiplex RT PCR assay are also comparable and in concordance with results from whole genome sequencing and VRE DNA microarray experiments [3] using the same VRE strains. Therefore, we have an accurate, fast, and economical method that can be used for quick diagnosis and patient screening for VRE from cultured colonies (without the need for genomic DNA extraction) in hospitals, care homes, routine diagnostic laboratories, etc. The data obtained from this study are also transferable for use in molecular point-of-care platforms, and can be expanded with more target genes and/or adapted to other pathogens. The assay can also be employed in development of ready-to-use qPCR VRE LyoBeads, and, as future work, it can be transferred to a system with no multiplexing limitations, like the BLINK system [45].

Multiplex RT PCR assays have been developed, and are still being developed, for various pathogens, including VRE. Multiplex RT PCR’s optimization process commences from the target selection, primer design, DNA isolation method, and careful and systematic optimization, through testing and evaluation, of the concentration of biochemical substances and parameters like annealing temperature, number of cycles, time of cycles, etc. The differences between multiplex RT PCR methodologies can be seen within these optimizations. For example, in one study, the sample used was direct fecal material, and the method included PCR primers and fluorescence resonance energy transfer hybridization probes specific to *vanA* and *vanB* [46]. In a second similar study, species-specific markers for enterococcus were not included, although *vanM* and an “internal control” [37] target for enterococcus was included. The sample material was also obtained from rectal swabs [37]. In the present study, markers for the detection of *Enterococcus faecalis* and *Enterococcus faecium* were included (so it was possible to detect cultures with a mix of both species), along with *vanA* and *vanB* resistance markers. Heat lysis was used for DNA isolation from cultured VRE strains. There are few differences among these methodologies; however, differences in performance between different multiplex RT PCR assays can only be determined with a parallel experiment.

RPA was also used in this study, as a method for the verification of the multiplex PCR results, and it has the advantage that the amplification reactions are initiated quickly, at a constant temperature, and without the need for special instruments. The RPA assay works, and the optimized protocols can be used in developing a new molecular isothermal point-of-care platform, or can be transferred to an existing one. It also offers another possible method for patient screening for VRE, as well as for use in routine diagnostic laboratories.

Though the main objective of this study was achieved, there are some limitations to be considered regarding the multiplex PCR technique for VRE. Firstly, only the specific four targets can be amplified in one run, meaning that the possible presence of other vancomycin/glycopeptide resistance genes will not be detected. Secondly, there is the possibility of preferential amplification of certain targets, because the presence of two or more primer pairs in multiplex PCR increases the chance of obtaining false amplification products, mainly because of the formation of primer dimers. These non-specific products may be more readily amplified than the intended target, thereby consuming reaction substances and resulting in poor annealing and extension rates [42,43]. Non-specific interactions can be minimized by paying attention to primer design parameters, such as the homology of primers with their target nucleic acid sequences, their length, their GC content, and their concentration. It should also be noted that a PCR run can be contaminated [31].

RPA has the disadvantage that the reagents needed are still comparably expensive, with only a few commercially available kits. RPA is prone to non-specific amplification, and it can also be easily contaminated [33,38,47]. RPA multiplex reactions are challenging, because the primers often form primer dimers [47]. The RPA primers in this study were not multiplex, because this was not the main aim of the study. In summary, a multiplex real-time PCR assay was optimized for VRE, and it was found that the results can be obtained quickly, within two hours, from cultured isolates.

## 4. Materials and Methods

### 4.1. RPA Oligonucleotides

*E. faecium (ddl)*, *vanA*, and *vanB* primers for RPA were obtained from a published paper [48]. *E. faecalis (rpoA)* oligonucleotides for RPA were obtained from another published paper [49] (see Table 5).

### 4.2. Samples

Thirty-eight vancomycin-resistant enterococcal strains used in this study were obtained from the University Hospital Regensburg, Germany, and two were obtained from the University Hospital Jena, Germany. The isolates originated from urine, blood, respiratory material, ascites, and swabs, and have been characterized in a previous study using whole genome sequencing and DNA microarray for VRE [3]. Thirty-four of the strains were *Enterococcus faecium* and six were *Enterococcus faecalis*. An overview of these reference VRE strains can be seen in Supplemental File S4.

### 4.3. Fast Lysis Protocol Without Purification

Two full 5 µL sterile inoculation loops of the cultured VRE strains were added to 100 µL phosphate buffered saline (Invitrogen PBS, fisher scientific, Schwerte, Germany) in a 1.5 µL Eppendorf tube (Eppendorf AG, Hamburg, Germany). The tube was then placed on a shaker, Bioshake IQ (QINSTRUMENTS, Jena, Germany), at 99 °C for 10 min. Afterwards, the tube and its contents were centrifuged at 14,000 rpm for 5 min; then, 50 µL of the supernatant was carefully pipetted into a new tube for PCR.

### 4.4. DNA Dilutions

DNA dilutions for the multiplex PCR and RPA assays were in the ratio 1:10, over seven dilutions. Each dilution was increasingly diluted by 10, until the seventh dilution. The first dilution was based on the stock DNA concentration in ng/µL and the size of the strain’s genome.

Number of genomes/µL = [DNA concentration (ng/µL) * 6.022 * 10^23^ (mol^−1^)]/[genome size (bp) * 1 * 10^9^ (ng/g) * 650 (g/mol)]

(Avogadro’s constant = 6.022 * 10^23^ (mol^−1^)]; correction factor = 1 * 10^9^ (ng/g); DNA weight = 650 (g/mol)).

DNA concentrations were measured using an Invitrogen Qubit 4 Fluorometer (Fischer scientific GmbH, Schwerte, Germany).

### 4.5. Multiplex PCR

The QuantStudio 5 (applied biosystems by Thermo Fisher scientific, Bremen, Germany) with 96 wells was used for all the real-time PCR experiments. Template DNA was used in 10-fold serial dilutions (D2-D7, i.e., 1 * 10^6^ genomes/µL–10 genomes/µL). A master mix was made in the bench for each experiment, adding up to 21 reactions (6 different dilutions repeated 3 times, with a negative control for each repetition). The total volume in each well was 20 µL, consisting of master mix (12 µL) and DNA template (8 µL). The estimated pipette volume error was 5%. The calculated concentrations for the master mix can be seen in Table 6. The PCR program in QuantStudio 5 was set for 40 cycles of the following three steps: step 1, 300 s at 95 °C; step 2, 20 s at 95 °C; step 3, 20 s at 54 °C. The PCR cycle number was set to 40 to achieve optimal yield and reduce non-specific products. Also, the available resources would be depleted beyond this point. The multiplex PCR results were accessible and easy to read using the QuantStudio^TM^ Design andAnalysis Software v1.5.1 (applied biosystems by Thermo Fisher scientific, Bremen, Germany).

### 4.6. RPA

Recombinase polymerase amplification (RPA) experiments were also carried out using QuantStudio 5 (applied biosystems) with 96 wells. Template DNA samples were selected from 10-fold serial dilutions (D2–D7). The fourth and fifth dilutions were used for the experiments [D5 (1000 genomes/µL) for *rpoA_faecalis* and D4 (10,000 genomes/µL) to test for the other targets]. A master mix was created by combining the primers, water, buffer, RPA exo Lyobeads (biotechrabbit GmbH, Berlin, Germany) (see Table 7), and sample DNA in a micro 0.2 mL PCR tube (Sigma-Aldrich, Taufkirchen, Germany), and initiator was added to the lid of the tubes, which were briefly centrifuged; then, each PCR tube was quickly placed in the QuantStudio device. Amplification reactions were initiated as soon as the initiator came into contact with the mix. Each tube contained 48 µL of the master mix and 2 µL of DNA template for each reaction. A control experiment with no DNA was carried out alongside each RPA experiment.

The calculated concentrations for the master mix are provided in Table 8. The RPA exo LyoBeads (biotechrabbit GmbH) were reconstituted based on the buffer concentration, and designed for a reaction volume of 50 µL. The buffer used here had a double concentration (2×). The RPA program used in QuantStudio 5 was set to run for 90 cycles at a constant temperature of 42 °C. Thus, each RPA run was completed in 31.5 min.

### 4.7. Gel Electrophoresis

A volume of 10 µL of GelRed (Sigma-Aldrich) was added, along with 1.5 g of agarose gel (Sigma-Aldrich, Taufkirchen, Germany), to 100 mL of Tris-acetate-EDTA (TAE) buffer to make gels for gel electrophoresis. A TAE buffer was prepared by adding 2 mL of 50× TAE buffer to 98 mL of distilled water. The gels were used for gel electrophoresis of the amplification products from RPA. A standard was prepared with 2.5 µL buffer + 4 µL 1 kB standard, and the RPA products were prepared by adding 2.5 µL buffer to 7.5 µL of each RPA product. A Mupid-One (Nippon genetics Europe, Düren, Germany) was used for electrophoresis, with TAE buffer added to the attached tray. The prepared gel forms were placed in the tray containing the buffer solution, and the standard was pipetted onto the first row of the gel; the amplification products, alongside the controls, were then pipetted onto the gel accordingly. The gel electrophoresis run was set for 30 min at 100 volts, after which the gel was viewed under ultraviolet (UV) light using the VWR Imager CHEMI Premium (VWR International Ltd., Leicestershire, UK). Images of the gel were taken to determine whether RPA products were present or not, and to assess their sizes.

## Figures and Tables

**Table 1:** List of primers and probes for multiplex PCR. Primer_left_0_sequence_fwd (5′-3′) denotes forward primer, primer_right_0_sequence_revcomp (5′-3′) denotes reverse complement, and probe_internal_0_sequence denotes probe.

Target	Primer–Probe Set	Sequence (5′-3′)
*ddl_faecalis*	primer_left_0_sequence_fwd (5′-3′)	AAGTAGCCATTTTAGGAAAT
	primer_right_0_sequence_revcomp (5′-3′)	GCATCATAATCATAGAAAGC
	probe_internal_0_sequence	CCGTACGACTTTACCTGGTGAAGTGG
*ddl_faecium*	primer_left_0_sequence_fwd (5′-3′)	ACATTGAATATGCCTTATGT
	primer_right_0_sequence_revcomp (5′-3′)	TTGGTCATGATTTTATCCAT
	probe_internal_0_sequence	CAGGCGTATTGACCAGTGCATGTG
*vanA*	primer_left_0_sequence_fwd (5′-3′)	CATGTTGATGTAGCATTTTC
	primer_right_0_sequence_revcomp (5′-3′)	AATTCAAACAGACCTTGTAT
	probe_internal_0_sequence	GGCAAGTCAGGTGAAGATGGATCC
*vanB*	primer_left_0_sequence_fwd (5′-3′)	TTGCTCGGAGGAACATGAT
	primer_right_0_sequence_revcomp (5′-3′)	TCTTGCATAGCTTCCATA
	probe_internal_0_sequence	GAAAAATTCGATCCGCACTACATCGG

“0” denotes first primer and probe combination chosen from ten predicted primer–probe pairs.

**Table 2:** Results of calibration curves for *vanA*, *vanB*, *ddl_faecalis*, and *ddl_faecium*.

Target	VRE Strains	Dye	Lift Δ Rn (Mean) D5	Calibration Curve Efficiency
*vanA*	95735_UK040	Cy5	1,150,000	98
*vanB*	95738_UK043	ATTO 550	600,000	95
*ddl_faecalis*	95737_UK045	FAM	827,212	94
*ddl_faecium*	95738_UK043	ROX	547,649	90

**Table 3:** List of VRE strains and accompanying multiplex PCR and RPA results, showing whether any of specified targets are present or absent in each strain.

	Multiplex RT PCR				RPA			
Strains ID	*ddl_faecalis*	*ddl_faecium*	*vanA*	*vanB*	*rpoA_faecalis*	*ddl_faecium*	*vanA*	*vanB*
97629	N	P	P	N	N	P	P	N
97903	N	P	P	N	-	P	P	N
97718	N	P	P	N	N	P	P	N
97618	N	P	N	P	N	P	-	P
97721	N	P	N	P	-	P	N	P
97728	N	P	N	P	-	P	N	P
97731	N	P	N	P	-	P	N	P
97778	N	P	N	P	N	P	N	P
97875	N	P	P	N	-	P	P	-
97878	N	P	P	N	-	P	P	-
97914	N	P	P	N	-	P	P	-
97631	P	N	P	N	P	-	P	-
97635	P	N	P	N	P	-	P	-
97636	P	N	P	N	P	N	P	-
97633	P	N	P	N	P	N	P	-
97643	P	N	P	N	P	N	P	-
97644	P	N	P	N	P	N	P	-
97880	N	P	P	N	-	P	P	-
97889	N	P	P	N	-	P	P	-
97925	N	P	N	P	-	P	-	P

P—positive (target present), N—negative (target not present), (-)—not tested.

**Table 4:** Differences between multiplex PCR assay, RPA assay, whole genome sequencing (WGS), and VRE DNA microarray for detection and/or characterization of VRE isolates.

Parameters	*Multiplex RT PCR*	*RPA*	*WGS*	*VRE DNA Microarray*
Working time	Between 2 and 3 h [37].	About 20 min [38].	Between 7 and 9 h, starting from VRE cultures [39]. In real-life clinical settings, time between sample collection and result can range from 24 h to 33 days [40].	About 4–5 h, starting from VRE cultures [3].
Scope of application	Screening method/confirmation of susceptibility results.	Screening method/confirmation of susceptibility results.	Characterization of isolates.	Characterization of isolates.
Number of target genes detected	Four to six [37].	One.	Thousands, all that are present [41].	Multiple genes to more than 300 [3].
Disadvantages	Requires use of expensive equipment. Requires trained personnel. Possibility of false amplification products, because of formation of primer dimers, resulting in poor annealing and extension rates [42]. Prone to contamination. Only specified targets can be detected.	Only few commercially available kits. Not suitable for large-scale experiments. High possibility of non-specific amplification [38]. Prone to contamination. Only specified target can be detected.	Requires trained personnel to obtain, analyze, and correctly interpret results [40]. Restricted to specialized laboratories. Large dataset. Not suitable for quick screening.Relatively expensive. Requires expensive hardware and/or expensive reagents.	Requires minimal training. Only targets present on microarray can be detected. Compared to PCR methods, “proofreading” can be achieved by using multiple primers/probes per target or target operon.
Advantages	Quick sample preparation, reagents are relatively affordable, there are many commercially available kits for PCR. DNA quantity of 1–5 ng is sufficient [43]. Suitable for screening.	Does not require expensive cycling equipment. Short run time [44]. Suitable for screening.	Can detect new genes or genes that are not pre-defined in panel.	Affordable. More than 300 targets genes can be detected. Suitable for use in outbreaks, for detection and tracking of transmission chains.

**Table 5:** RPA primers and probes, including their sequences.

Target	Primer–Probe Set	Sequence (5′-3′)	Product Length
*ddl*	ddl-F	ACCCAAGTGGACAGACAGAGGAAGGCTTTA	156 bp
	ddl-R	TTCCATCTTCCCCGTTTGGCCCATGTAAAACT	
	ddl-F_revcomp	TAAAGCCTTCCTCTGTCTGTCCACTTGGGT	
	ddl-R_revcomp	AGTTTTACATGGGCCAAACGGGGAAGATGGAA	
*vanA*	vanA-F	TTGCGCGGAATGGGAAAACGACAATTGCTATT	194 bp
	vanA-R	CAAAAGGGATACCGGACAATTCAAACAGACC	
	vanA-F_revcomp	AATAGCAATTGTCGTTTTCCCATTCCGCGCAA	
	vanA-R_revcomp	GGTCTGTTTGAATTGTCCGGTATCCCTTTTG	
*vanB*	vanB-F	GAGGATGATTTGATTGTCGGCGAAGTGGAT	165 bp
	vanB-R	TTTGCCGTTTCTTGCACCCGATTTCGTTCCTC	
	vanB-F_revcomp	ATCCACTTCGCCGACAATCAAATCATCCTC	
	vanB-R_revcomp	GAGGAACGAAATCGGGTGCAAGAAACGGCAAA	
*rpoA*	rpoA-F	GGACCCGCTACCGTGACTGCCGGCGATATTATCG	197 bp
	rpoA-R	GAATCAACTGGAAGTACACCGATTGGCATATC	
	rpoA-F_revcomp	CGATAATATCGCCGGCAGTCACGGTAGCGGGTCC	
	rpoA-R_revcomp	GATATGCCAATCGGTGTACTTCCAGTTGATTC	

F—forward primers, R—reverse primers, revcomp—reverse complement, bp—base pairs.

**Table 6:** Substances contained in master mix and their calculated concentrations.

Substances	Unit	Final Concentration	Volume/Well (µL)
qPCR Buffer	mM	1	2.00
MgCl_2_	mM	3	1.20
dNTP/dUTP (BTR)	mM	0.2	0.40
ddl-faecalis_fwd_left_0	µM	0.2	0.04
ddl-faecium_fwd_left_0	µM	0.2	0.04
vanA_fwd_left_0	µM	0.2	0.04
vanB_fwd_left_0	µM	0.2	0.04
ddl-faecalis_revcomp_rigth_0	µM	0.2	0.04
ddl-faecium_revcomp_rigth_0	µM	0.2	0.04
vanA_revcomp_rigth_0	µM	0.2	0.04
vanB_revcomp_rigth_0	µM	0.2	0.04
ddl-faecalis_probe_0	µM	0.2	0.04
ddl-faecium_probe_0	µM	0.2	0.04
vanA_probe_0	µM	0.2	0.04
vanB_probe_0	µM	0.2	0.04
Polymerase (BTR)	U/µL	0.2	0.80
BSA (NEB)	mg/ml	1	1.00
Uracil-DNA Glycosylase (BTR)	U/µL	0.01	0.20
Template (DNA)	-	-	8.00
water, nuclease-free	-	-	5.92
Total volume	-	-	20.00

MgCl_2_—magnesium chloride, dNTP—deoxynucleoside triphosphate, dUTP—2′-Deoxyuridine, 5′-Triphosphate, BTR—biotechrabbit GmbH, Berlin, Germany, BSA—bovine serum albumin, NEB—New England biolabs, Ipswich, MA, USA.

**Table 7:** Buffer volume and concentration contained in one RPA LyoBead for one reaction.

Components	Concentration		
	Stock	Reaction	Reaction
			1 bead
Water (H_2_O)			17.80 µL
RPA reconstitution buffer 10	2×	1.17	25.00 µL
Total volume			42.8 µL

Note: LyoBead is designed for 50 µL reaction. Volume of H_2_O (water) can be variable.

**Table 8:** All components for RPA and their concentrations, including DNA template.

Components	Concentration		
	Stock	Reaction	
Reconstituted LyoBead RPA	1.17×	1×	42.8 µL
Volume of pre-mix			43.5 µL
Template (DNA)			2.0 µL
fw_Primer	20 µM	0.2 µM	0.5 µL
rev_Primer	20 µM	0.2 µM	0.5 µL
EvaGreen (dye)	50×	1×	1.0 µL
RPA reaction initiator	20×	1×	2.5 µL
Total volume per reaction			50.0 µL

## Data Availability

The datasets generated for this study are available on request to the corresponding author.

## Supplementary Materials

The following supporting information can be downloaded at https://www.mdpi.com/article/10.3390/antibiotics14030295/s1: Supplemental File S1: PCR calibration curve data for all targets; Supplemental File S2: Multiplex PCR results for VRE strains, including diagnostic sensitivity and specificity; Supplemental File S3: Gel electrophoresis images of RPA—VRE experiments; Supplemental File S4: Reference strains used in study.
