# Supplementary material for: Multiplex Real-Time Polymerase Chain Reaction and Recombinase Polymerase Amplification: Methods for Quick and Cost-Effective Detection of Vancomycin-Resistant Enterococci (VRE)
Source: Antibiotics (Basel). 2025 Mar 12;14(3):295. doi: 10.3390/antibiotics14030295 (PMC11939700; doi:10.3390/antibiotics14030295)
Supplement: Supplementary file 1 [file antibiotics-14-00295-s001.zip › Supplemental File S3.docx]

**RPA-VRE Gel Results**

**1) Positive Targets**

C - control


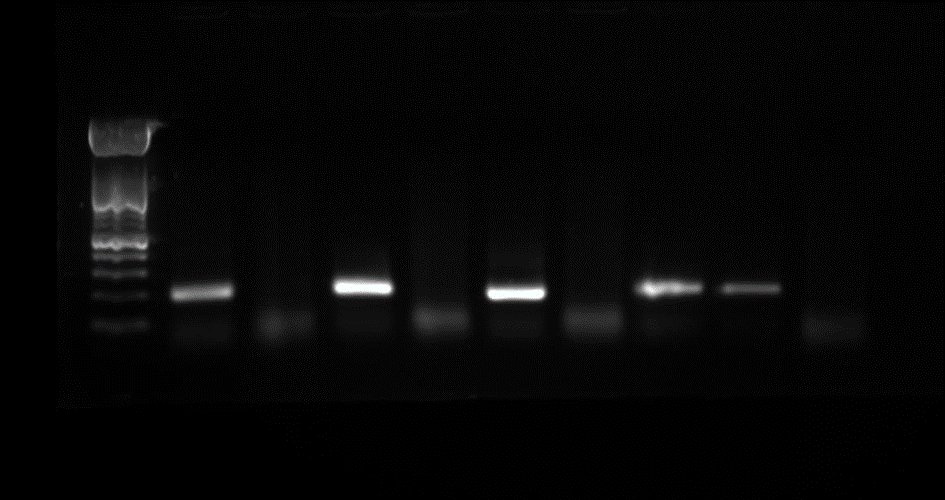


C

97903

97903

C

97633

C

97631

Con

DNA ladder

**A**

*vanA*

97629

*1.5kbp*

*50bp*

Figure A: Gel electrophoresis image of RPA product for two different *vanA*-positive *Enterococcus faecalis* (97631, 97633), two *vanA*-positive *Enterococcus faecium* (97629, 97903) and the control experiments (C). *vanA* was amplified in all four strains as shown by the presence of an amplification product corresponding to about two hundred base pairs (see arrow).


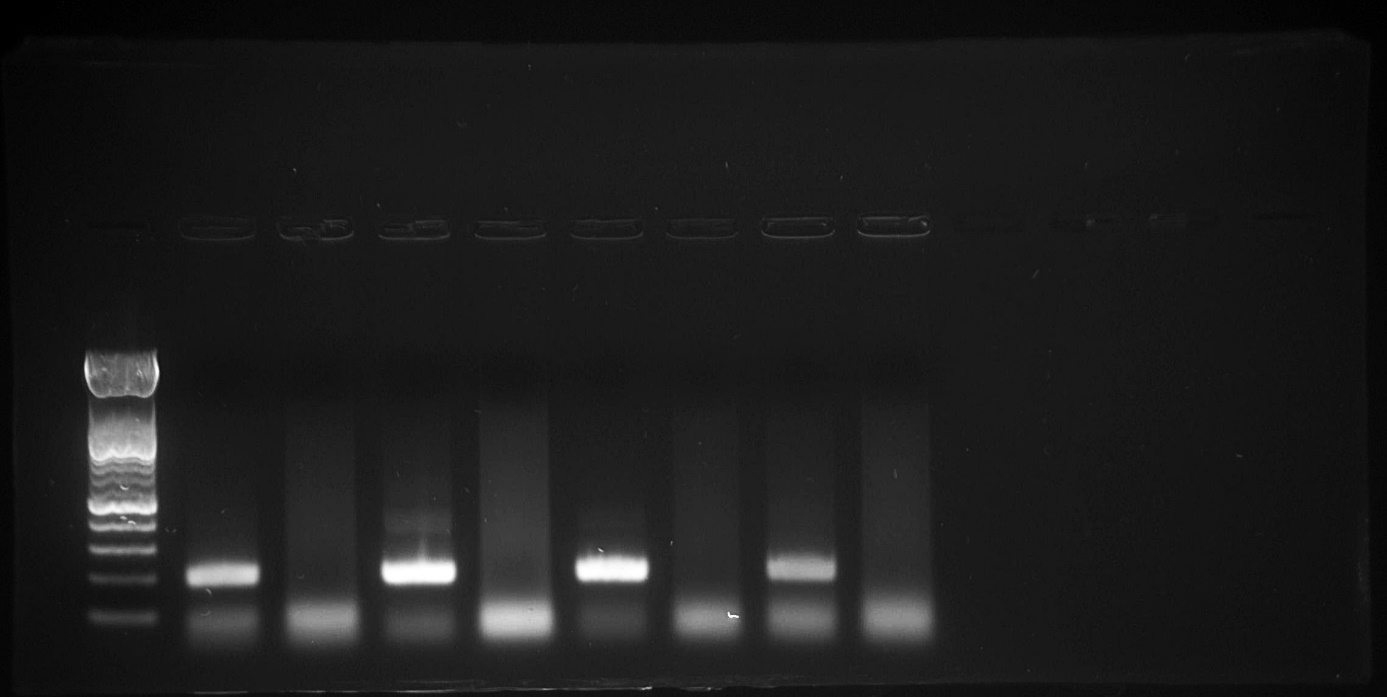


DNA ladder

C

97636

C

97643

C

97644

C

97914

**B**

*vanA*

*50bp*

*1.5kbp*

Gel electrophoresis image of RPA product for three different *vanA*-positive *Enterococcus faecalis* (97636, 97643, 97644), one *vanA*-positive *Enterococcus faecium* (97914) and the control experiments (C). *vanA* was amplified in all four strains as shown by the presence of an amplification product corresponding to about two hundred base pairs (see arrow).


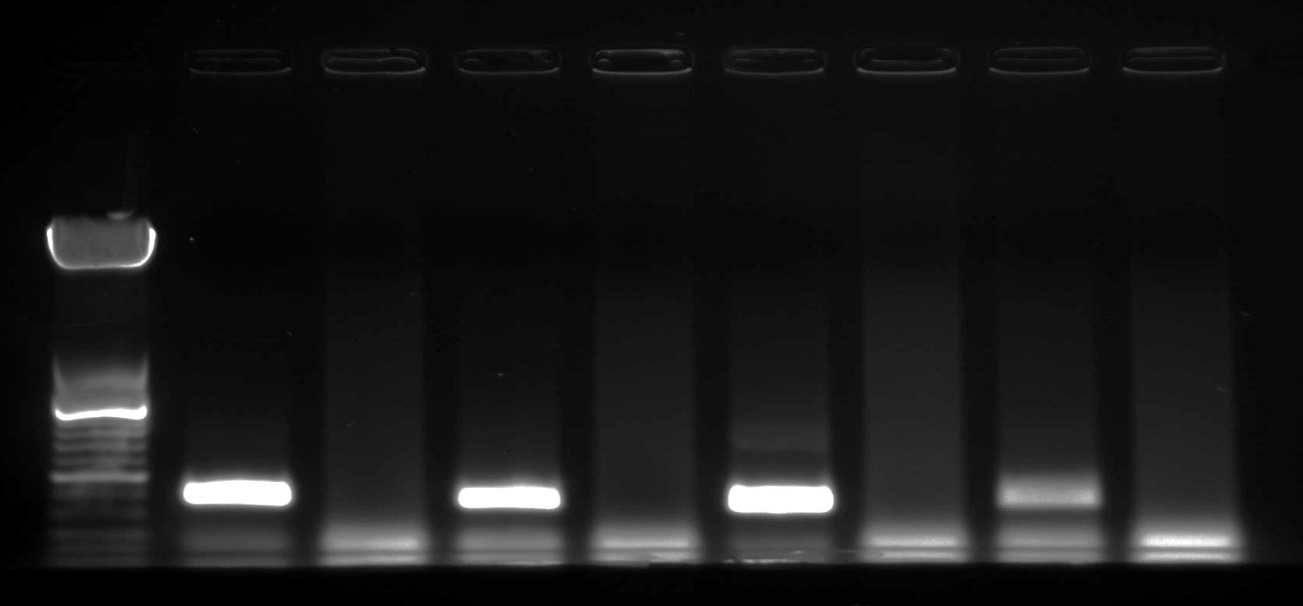


*50bp*

**C**

*1.5kbp*

C

C

C

C

DNA ladder

97875

97880

97878

97718

*vanA*

Figure C: Gel electrophoresis image of RPA product for four different *vanA*-positive *Enterococcus faecium* (97875, 97718, 97878 and 97880) and the control experiments (C). *vanA* was amplified in all four strains as shown by the presence of an amplification product corresponding to about two hundred base pairs (see arrow).


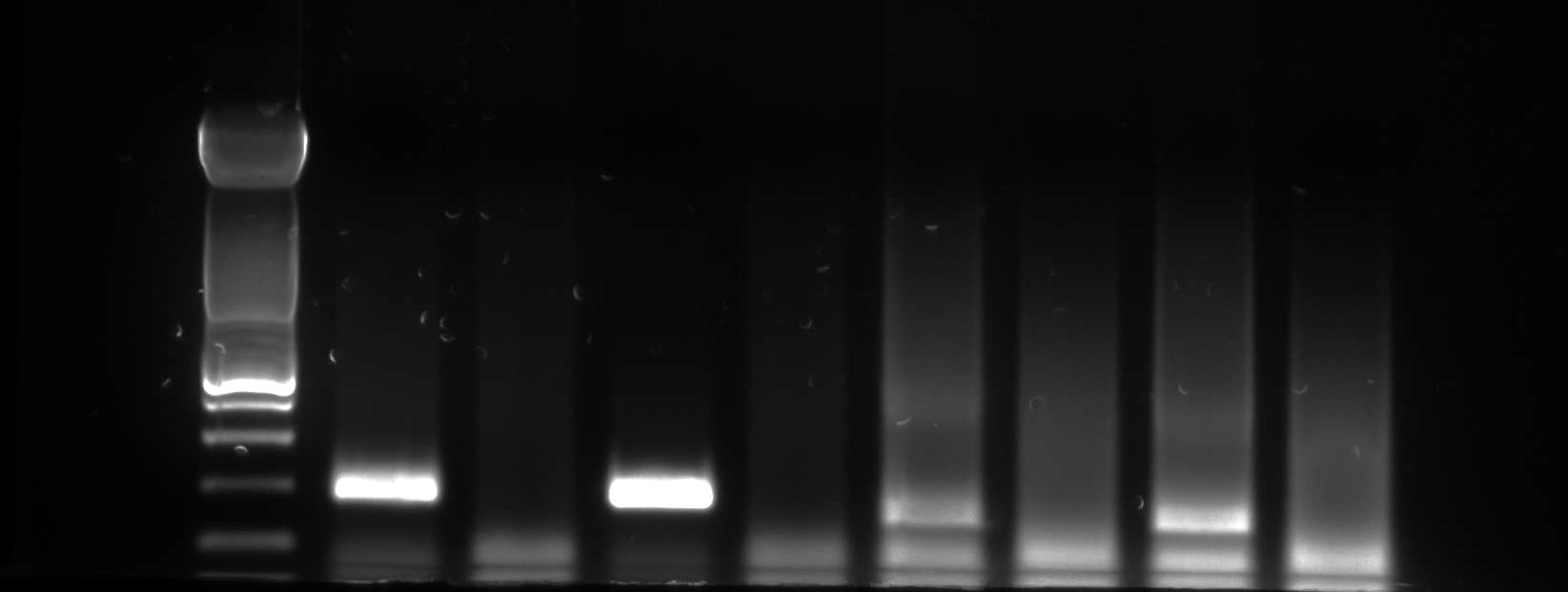


C

97721

C

97618

C

97635

C

97889

*50bp*

*<*

DNA ladder

**D**

*1.5kbp*

*vanB*

*vanA*

Figure D: Gel electrophoresis image of RPA product for one *vanA*-positive *Enterococcus faecium* (97889), one *vanA*-positive *Enterococcus faecalis* (97635). On the right side of the image is RPA product for two *vanB*-positive Enterococus faecium (97618 and 97721). Control experiments are marked (C). *vanA* was amplified in two strains as shown by the presence of an amplification product corresponding to about two hundred base pairs (see blue arrow) and *vanB* was amplified in two strains as shown by the presence of amplification product in about one hundred and fifty base pair (see red arrow).


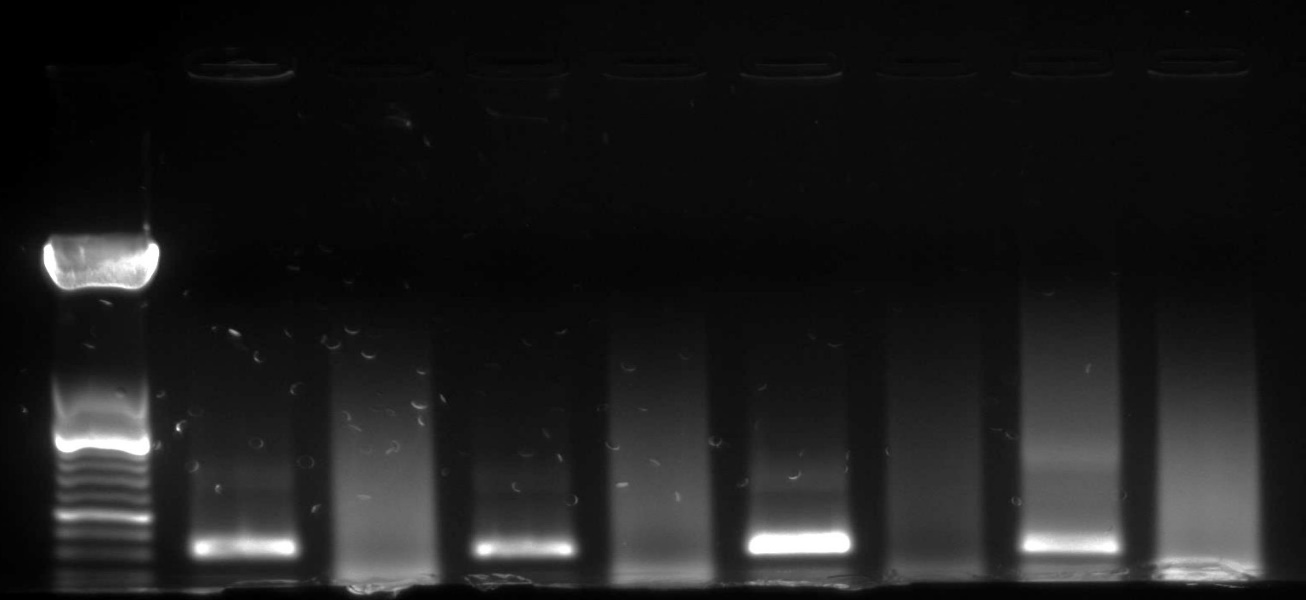


*vanB*

**E**

C

97778

C

97925

C

*50bp*

*<*

*1.5kbp*

DNA ladder

97728

C

97731

Figure E: Gel electrophoresis image of RPA product for four different *vanB*-positive *Enterococcus faecium* (97728, 97731, 97778, 97925) and the control experiments (C). *vanB* was amplified in all four strains as shown by the presence of an amplification product corresponding to about one hundred and fifty base pairs (see arrow).


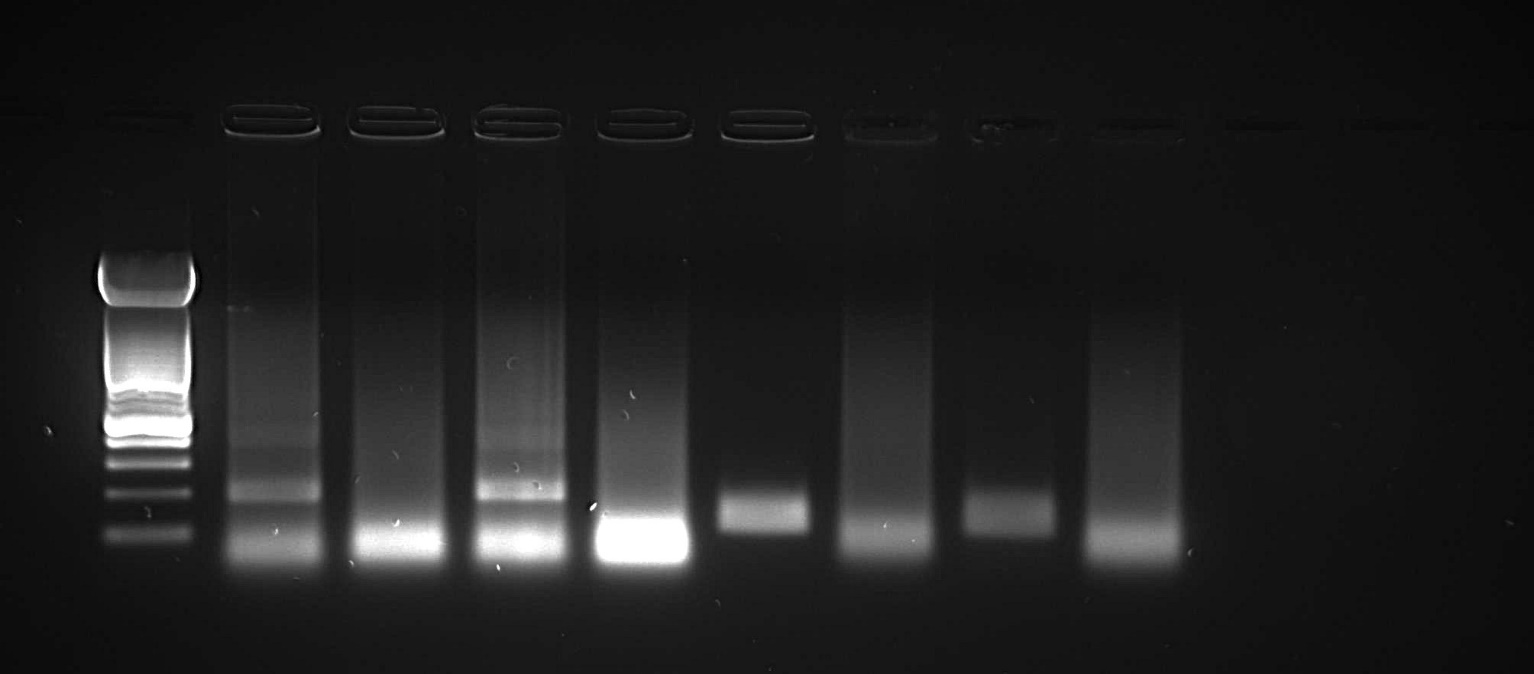


**F**

*ddl_faecium*

*rpoA_faecalis*

97925

C

C

97889

C

97631

*1.5kbp*

DNA ladder

*50bp*

*<*

C

97635

Figure F: Gel electrophoresis image of RPA product for two *rpoA_faecalis*-positive *Enterococcus faecalis* (97631 and 97635). On the right side an image of the RPA product for two *ddl_faecium*-positive *Enterococus faecium* (97889 and 97925). Control experiments are marked (C). *rpoA_faecalis* was amplified in two strains as shown by the presence of an amplification product corresponding to about two hundred base pairs (see blue arrow) and *ddl_faecium* was amplified in two strains as shown by the presence of amplification product in about one hundred and fifty base pair (see red arrow).


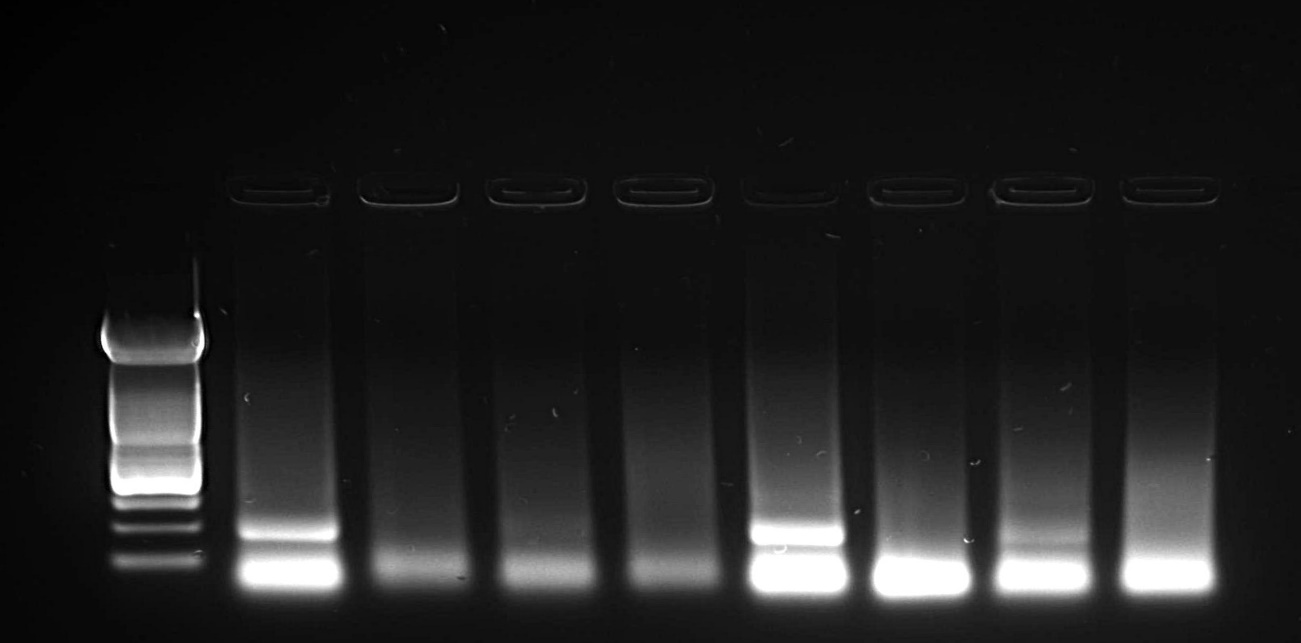


**G**

*1.5kbp*

*50bp*

*<*

*rpoA_faecalis*

C

97644

C

97643

C

97633

C

97636

DNA ladder

Figure G: Gel electrophoresis image of RPA product for four different *rpoA_faecalis*-positive *Enterococcus faecalis* (97636, 97633, 97643 and 97644) and the control experiments (C). *rpoA_faecalis* was amplified in three strains as shown by the presence of an amplification product corresponding to about two hundred base pairs (see arrow). RPA reaction was repeated subsequently for 97633.


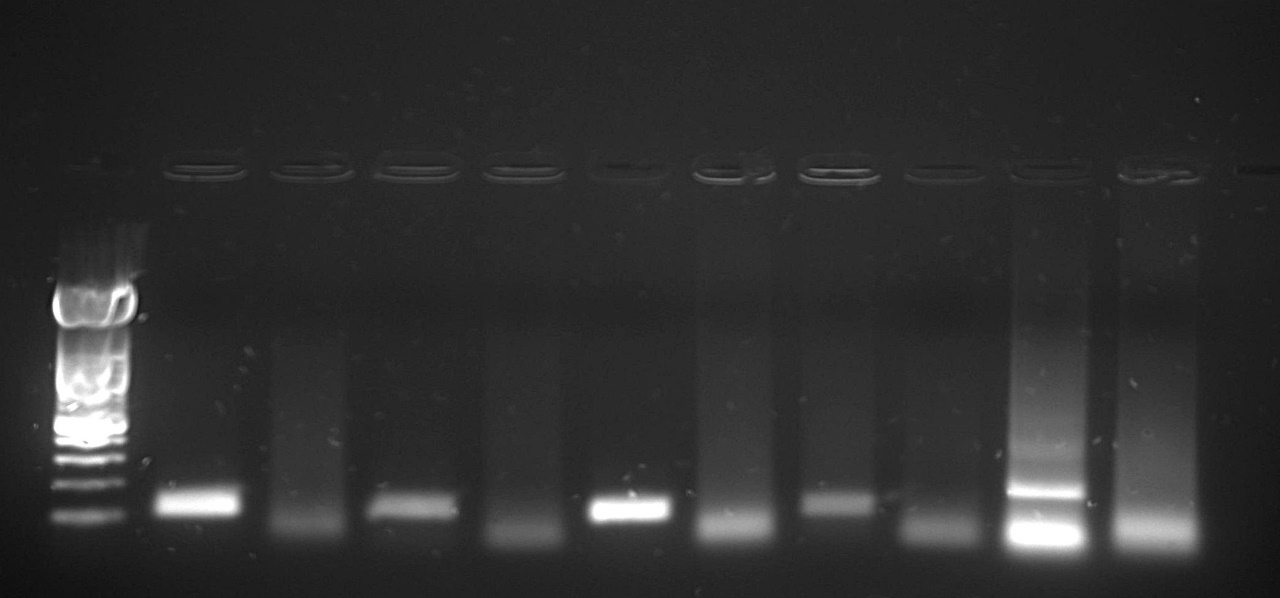


*rpoA_faecalis*

*ddl_faecium*

**H**

97629

97778

C

C

97618

C

97713

C

DNA ladder

97633

*50bp*

*<*

*1.5kbp*

C

Figure H: Gel electrophoresis image of RPA product for three *ddl_faecium*-positive *Enterococus faecium* (97629, 97713, 97618). On the right side the image of RPA product for one *rpoA_faecalis*-positive *Enterococcus faecalis* (97633). Control experiments are marked (C). *ddl_faecium* was amplified in three strains as shown by the presence of an amplification product corresponding to about one hundred and fifty base pair (see red arrow) and *rpoA_faecalis* was amplified in one strain as shown by the presence of amplification product in about two hundred base pairs (see blue arrow).


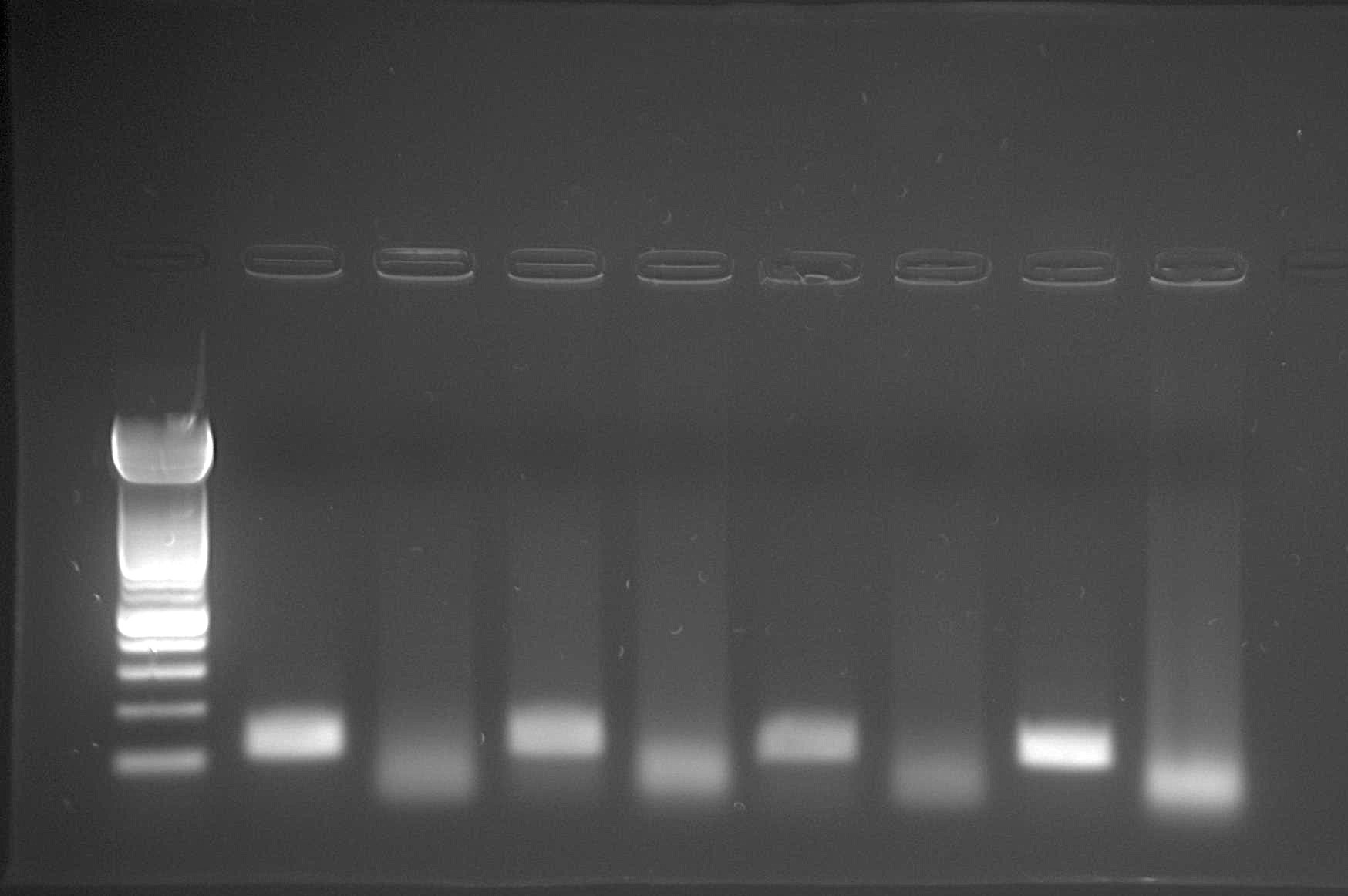


*50bp*

*<*

*ddl_faecium*

**I**

*1.5kbp*

C

97728

C

97718

C

97721

C

97731

DNA ladder

Figure I: Gel electrophoresis image of RPA product for four *ddl_faecium*-positive *Enterococus faecium* (97731, 97728, 97718 and 97721) and the control experiments (C). *ddl_faecium* was amplified in all four strains as shown by the presence of an amplification product corresponding to about one hundred and fifty base pair (see arrow).


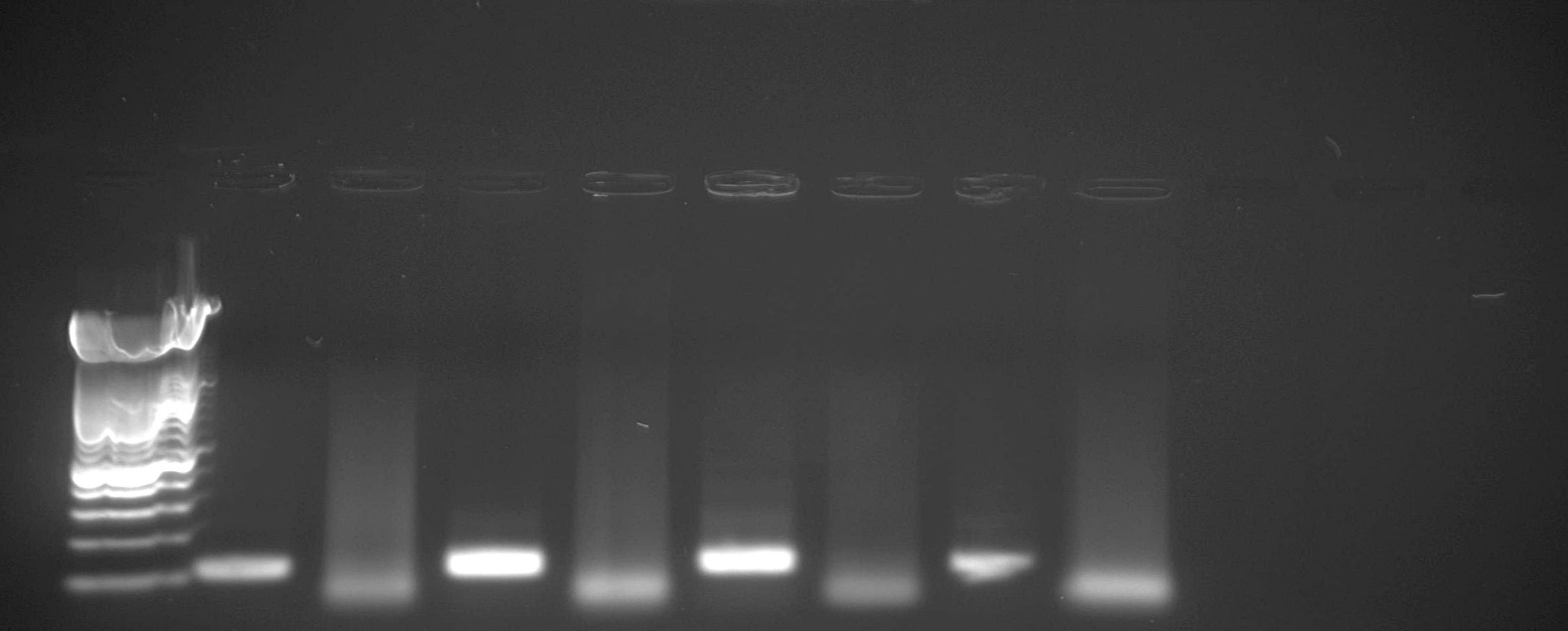


*50bp*

**J**

DNA ladder

*ddl_faecium*

97875

C

97878

C

97914

C

97880

C

*1.5kbp*

Figure J: Gel electrophoresis image of RPA product for four *ddl_faecium*-positive *Enterococus faecium* (97875, 97878, 97914 and 97880) and the control experiments (C). *ddl_faecium* was amplified in all four strains as shown by the presence of an amplification product corresponding to about one hundred and fifty base pair (see arrow).

**2) Negative targets**

C – control


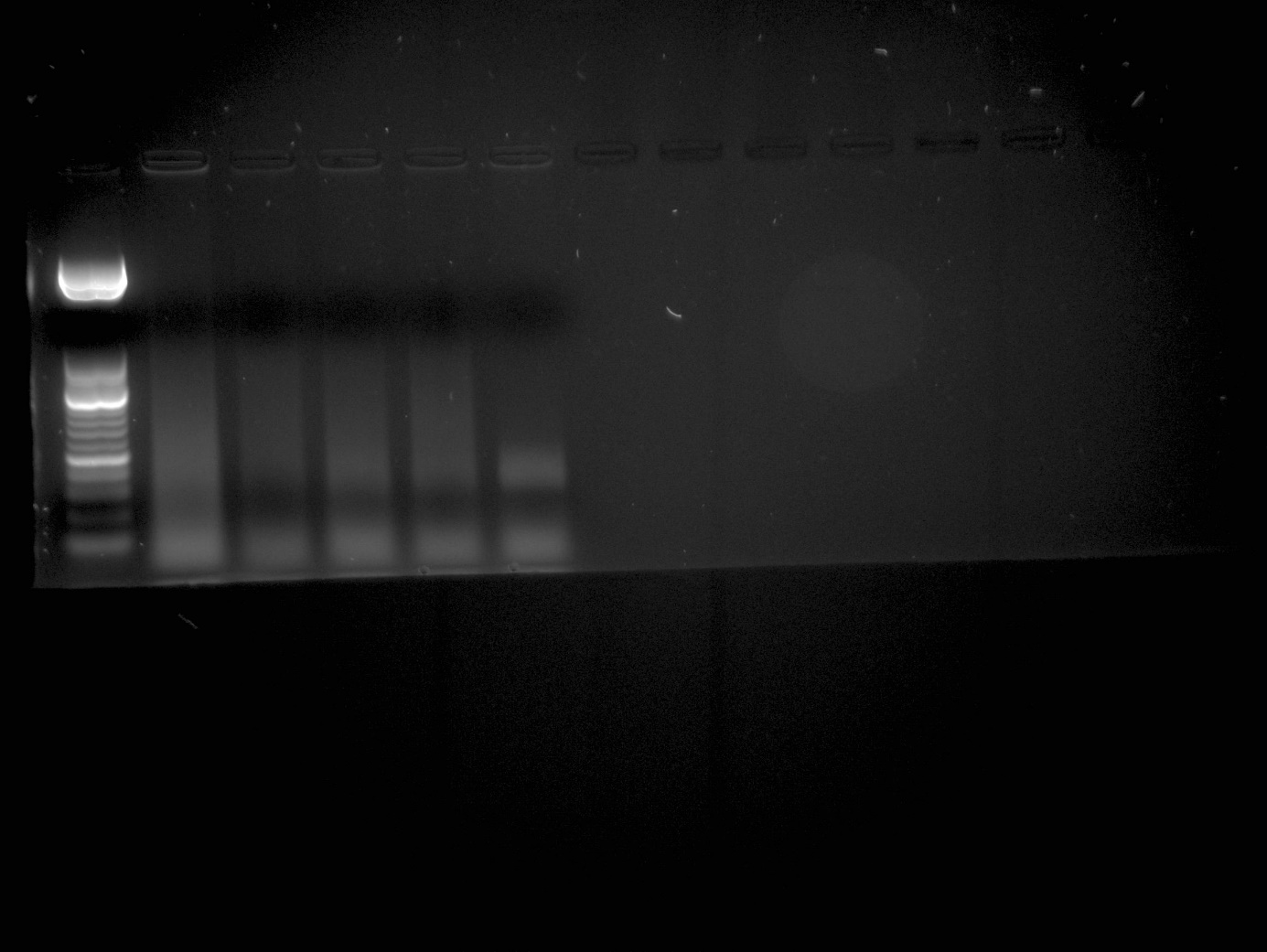


**K**

*vanB*

C

97718

C

97903

C

97629

DNA ladder

*50bp*

*1.5kbp*

Figure K: Gel electrophoresis image of RPA product for three different *vanB*-negative *Enterococcus faecium* (97629, 97903, 97718) and the control experiments (C). *vanB* was not amplified in all three strains.


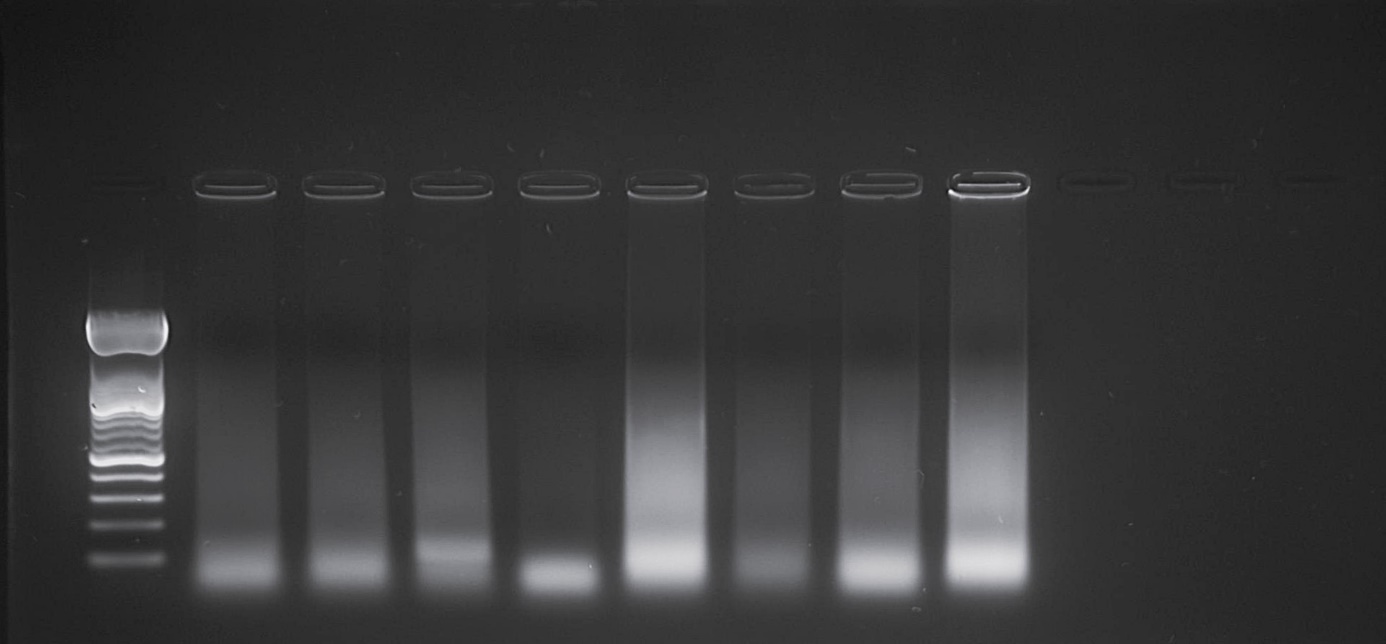


**L**

*50bp*

*<*

*1.5kbp*

97778

DNA ladder

97721

C

97728

97731

C

C

*vanA*

C

Figure L: Gel electrophoresis image of RPA product for four different *vanA*-negative *Enterococcus faecium* (97721, 97728, 97778, 97731) and the control experiments (C). *vanA* was not amplified in all four strains.


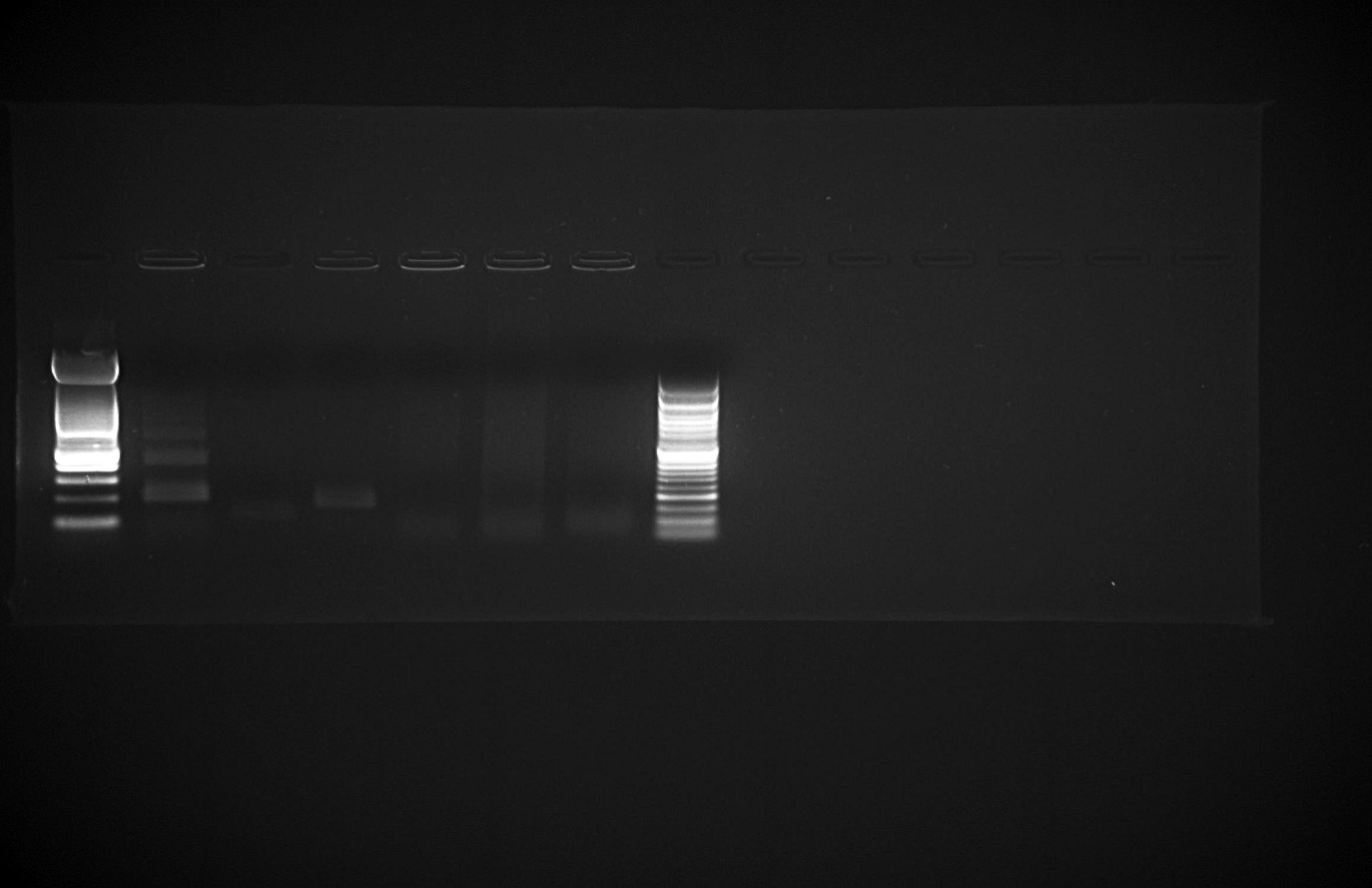


*rpoA_faecalis*

DNA ladder (100bp)

DNA ladder (50bp)

C

97629

C

97718

C

97778

*1.5kbp*

*50bp*

*<*

Figure M: Gel electrophoresis image of RPA product for three different *Enterococcus faecium* (97629, 97718, 97778) and the control experiments (C). *rpoA_faecalis* was not amplified in all three strains.

*rpoA_faecalis*


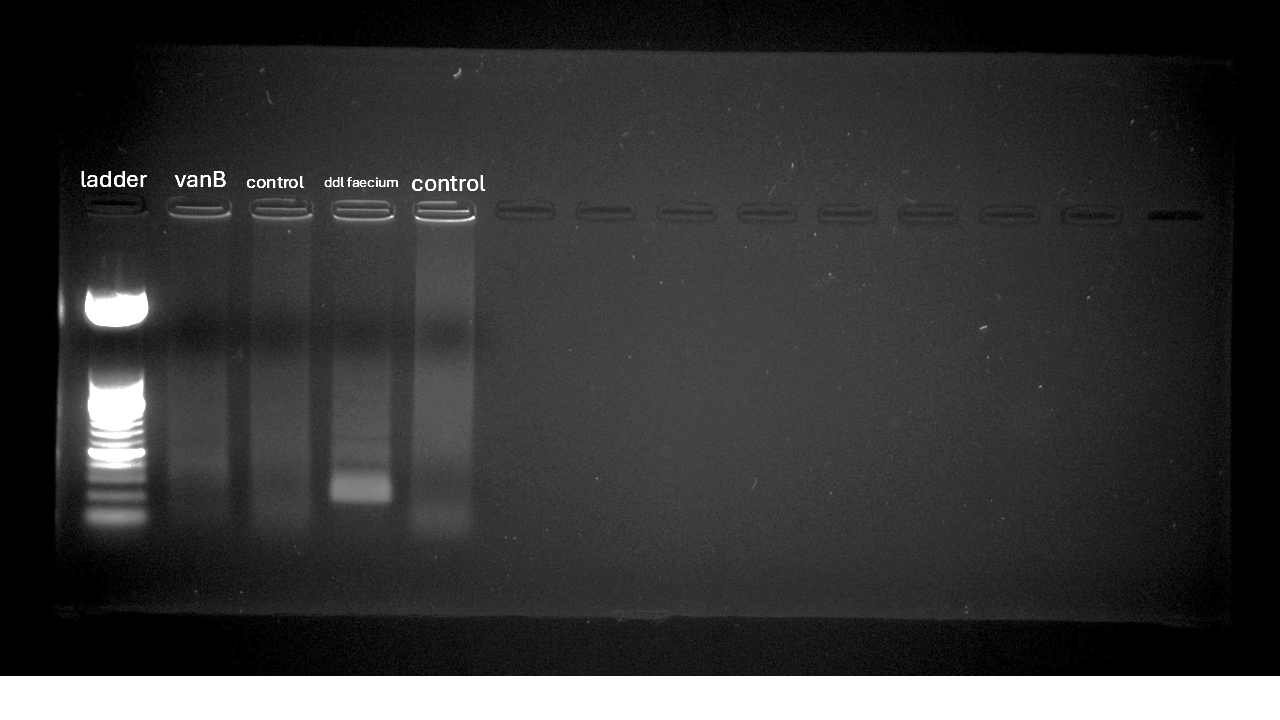


*1.5kbp*

*50bp*

*<*

97633 (rpoA positive)

DNA ladder (50bp)

C

C

97618

Figure N: Gel electrophoresis image of RPA product for one *Enterococcus faecium* (97618) and the control experiments (C). *rpoA_faecalis* was not amplified in the strain. Next to that is a repeat RPA reaction for *rpoA*-faecalis in *Enterococcus faecalis* (97633) and the gel product image is positive as seen by the presence of amplification product in about two hundred base pairs (see arrow).


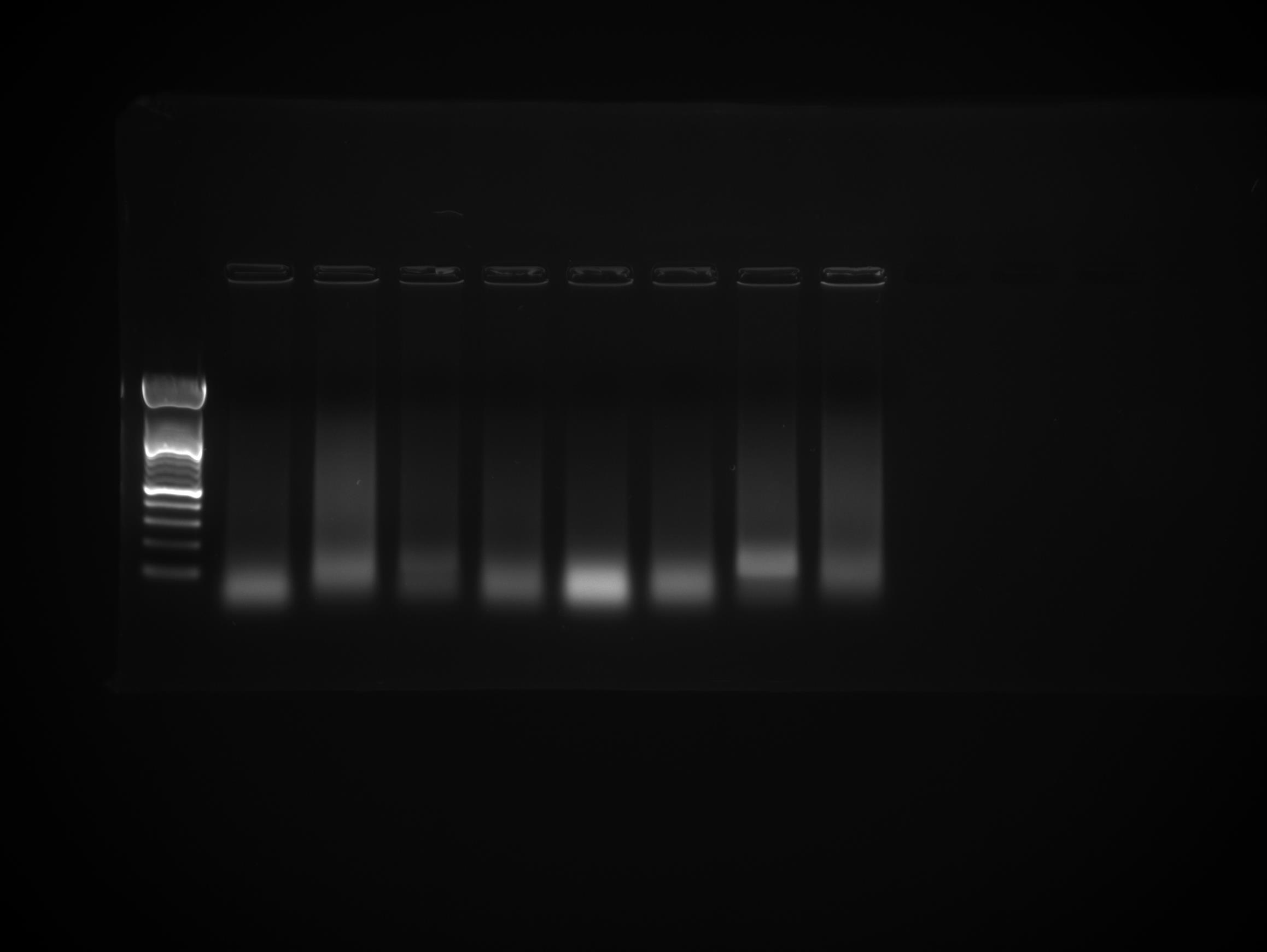


*ddl_faecium*

*50bp*

*<*

*1.5kbp*

97643

DNA ladder

C

97644

C

C

97633

C

97636

Figure O: Gel electrophoresis image of RPA product for four different *Enterococcus faecalis* (97636, 97633, 97643, 97644) and the control experiments (C). *ddl_faecium* was not amplified in all four strains.
